# Evaluation of Payer Policies to Reduce Low-Value Medical Device–Based Procedure Use

**DOI:** 10.1001/jamahealthforum.2025.3898

**Published:** 2025-10-31

**Authors:** Sanket S. Dhruva, Sarah R. Tingley, Michael Incze, John M. Neuhaus, Marcus A. Bachhuber, Rita F. Redberg

**Affiliations:** 1 University of California, San Francisco School of Medicine, San Francisco; 2 Philip R. Lee Institute for Health Policy Studies, University of California, San Francisco, San Francisco; 3 Section of Cardiology, Department of Medicine, San Francisco Veterans Affairs Medical Center, San Francisco, California; 4 Division of Cardiology, Department of Medicine, University of California, San Francisco School of Medicine, San Francisco; 5 Division of General Internal Medicine, Department of Medicine, University of Utah School of Medicine, Salt Lake City; 6 Department of Epidemiology and Biostatistics, University of California, San Francisco, San Francisco; 7 Department of Medicine, Oregon Health & Science University, Portland

## Abstract

**Importance:**

Low-value care occurs when the harms or costs of care exceed the benefits. Such care includes when medical device-based procedures are favored over medical management, such as invasive coronary angiography (ICA) and percutaneous coronary intervention (PCI) for stable coronary artery disease; endovascular intervention for lower extremity peripheral arterial disease with intermittent claudication; and nasal endoscopy with balloon ostial dilation and functional endoscopic sinus surgeries (collectively, sinus procedures) for chronic rhinosinusitis.

**Objective:**

To evaluate the association of enacting evidence-based clinical coverage policies for these low-value procedures with their use in Louisiana’s Medicaid program.

**Design, Setting, and Participants:**

Louisiana Medicaid partnered with academic investigators to develop and enact evidence-based policies between December 2021 and February 2022. This study used an interrupted time series approach to evaluate use of the 4 identified procedures 12 months before and 18 months after policy enactment. For each procedure, best-fit curves were constructed of prepolicy utilization trends, which were used to generate expected postpolicy trends. These expected trends were then compared to observed postpolicy utilization. Three-way analysis (time × intervention period [pre or post] × procedure) was used to assess changes in utilization slope for each procedure relative to colonoscopy (which had no policy changes and was expected to show baseline use of procedures in Louisiana Medicaid). Final follow-up was in September 2023.

**Main Outcomes and Measures:**

Monthly outpatient procedural utilization per 100 000 Louisiana Medicaid members of ICA, PCI, endovascular intervention, and sinus procedures.

**Results:**

There were 1 396 629 Louisiana Medicaid members with data at 1 year before any policy enactment and 1 548 265 at final follow-up. Overall, 14 940 individuals (mean [SD] age, 43.5 [13.7] years; 53.0% female) underwent one of these procedures before policy enactment, and 20 882 (mean [SD] age, 43.3 [13.7] years; 52.6% female) after enactment. Differences in monthly outpatient procedure rates in the postenactment compared with the preenactment period per 100 000 members were as follows: ICA, 0.65 (95% CI, 0.06 to 1.23); PCI, 0.15 (95% CI, −0.01 to 0.31); endovascular intervention, −0.01 (95% CI, −0.12 to 0.10); and sinus procedures, −0.23 (95% CI, −1.61 to 1.15). There was no significant change in the 3-way time × intervention period × procedure vs colonoscopy interaction for any procedure.

**Conclusions and Relevance:**

In this quality improvement study, enactment of evidence-based clinical coverage policies for 4 common medical device-based procedures was not associated with a reduction in their use in Louisiana Medicaid. These findings suggest a need for more comprehensive strategies to improve evidence-based care for Medicaid through multipronged efforts that include education, policymaking, and value-based coverage implementation.

## Key Points

**Question:**

What is the association of enacting evidence-based coverage policies for procedures that are often performed for low-value indications with utilization in a state Medicaid program?

**Findings:**

This quality improvement study used data from 1.5 million Louisiana Medicaid members to evaluate use of procedures identified as low-value (invasive coronary angiography and percutaneous coronary intervention for stable coronary artery disease, endovascular intervention for lower extremity peripheral arterial disease with intermittent claudication, and nasal sinus procedures for chronic rhinosinusitis). Enactment of evidence-based coverage policies was not associated with reductions in use of these procedures.

**Meaning:**

These findings suggest that reduction of low-value procedure use in Medicaid requires a multipronged approach to address the multiple drivers of such care, in addition to the enactment of evidence-based coverage policies.

## Teaser

This quality improvement study evaluates the association of enacting evidence-based clinical coverage policies with use of low-value procedures in a state Medicaid program.

## Introduction

Low-value care is defined as care in which harms or costs outweigh benefits. It is rife in health care,^1^ wasting resources^2^ and leading to avoidable patient injuries.^3,4^ Medical devices often receive Food and Drug Administration marketing authorization with limited or no clinical testing^5,6,7,8^ and are key drivers of low-value care. The sharply higher reimbursement of procedures compared to medical therapy (ie, pharmacologic and/or nonprocedural treatments) in the US health care system’s primarily fee-for-service (FFS) model^9^ can create incentives to perform procedures when medical management would be at least equally effective. High spending for low-value procedures is particularly burdensome for state Medicaid agencies, which have limited budgets.^10^ By draining fixed budgets and resources, spending on low-value procedures interferes with states’ abilities to provide needed care.

Invasive coronary angiography (ICA) and percutaneous coronary intervention (PCI) for stable coronary artery disease (CAD) are examples of common, highly reimbursed medical device–based procedures. Multiple high-quality randomized clinical trials (RCTs) have found no clinical outcome benefits for PCI compared with medical therapy.^11,12,13,14^ Further, there is no symptomatic benefit among patients who are treated with optimal medical therapy compared with PCI.^15^ PCI has multiple risks, including periprocedural bleeding, kidney injury, stroke, and the need for dual antiplatelet therapy. Even though PCI should be reserved for situations in which medical management fails, it is often performed with little or no attempt at medical management.^16^ Overall, approximately 200 000 PCIs are performed for stable CAD annually in the US^17,18^ at a cost of more than $3 billion annually.^19^

Endovascular intervention for lower extremity peripheral arterial disease (PAD) with intermittent claudication is another low-value procedure. RCTs have shown that procedural treatments do not offer benefit compared with supervised exercise therapy and medical management.^20,21,22^ Clinical practice guidelines strongly recommend supervised exercise therapy and medical management as first-line treatment approaches.^20^ However, more than 40 000 endovascular interventions are performed for the Medicare FFS population annually for intermittent claudication at more than $3600 per procedure,^23^ while supervised exercise therapy was provided to fewer than 2% of Medicare beneficiaries and medical management remains underused.^24,25,26^ The time to intervention after diagnosis has also been decreasing and is most recently reported as less than 2 months, which suggests short, if any, attempts at supervised exercise and medical management.^27^ Further, endovascular intervention can often lead to patient harm, including a “treatment trap” in which an initial vascular procedure may lead to multiple additional procedures and even amputation.^28^

Finally, despite limited evidence of benefit compared with medical therapy, nasal endoscopy with balloon ostial dilation (balloon sinuplasty) and functional endoscopic sinus surgeries (collectively, sinus procedures) are commonly performed for chronic rhinosinusitis.^29^ Sinus procedures should be considered only for patients with ongoing symptoms refractory to optimal, guideline-directed medical therapy.^30,31^ However, medical therapy, such as saline nasal irrigation or nasal corticosteroids, are underused in this condition^32^ even though they are substantially less expensive than the average $1500 per nasal sinus endoscopy procedure.^33^

All of the procedures we studied should be reserved for patients with ongoing symptoms refractory to optimal, guideline-directed medical therapy. However, all of these procedures have been used inappropriately in recent years.^33,34,35^ There are multiple drivers of low-value procedural utilization, such as clinician and hospital financial interests as well as patient beliefs that more care is better care.^36^ Campaigns such as Choosing Wisely have had limited success in reducing low-value care.^37^ A potential strategy to curtail such low-value care is rigorous, evidence-based clinical coverage policymaking in which non–evidence-based use is not reimbursed.^38,39,40^ Accordingly, we worked with Louisiana Medicaid leadership and stakeholders to develop and enact coverage policies with this goal for ICA and PCI, endovascular intervention, and sinus procedures and assess the effectiveness of policy enactment.^3^

## Methods

This quality improvement study was deemed exempt by the University of California, San Francisco institutional review board as it was secondary research for which informed consent is not required. This study followed the Standards for Quality Improvement Reporting Excellence (SQUIRE) reporting guideline.

### Policy Context

We formed a novel academia-policymaker collaboration between researchers at the University of California, San Francisco who focus on studying and reducing low-value care and leadership at Louisiana Medicaid, with the shared goal of reducing low-value medical device–based procedures through evidence-based coverage policymaking.^3^ We identified potentially low-value procedures with high utilization and spending by using Louisiana Medicaid claims, with a focus on procedures that had potential for direct patient harm or starting cascades of low-value care. While we did not have access to medical records and therefore could not specifically assess overuse in Louisiana Medicaid, we targeted procedures with significant evidence of overuse in other settings. We reviewed existing clinical coverage policies as well as clinical practice guidelines and the peer-reviewed literature. The new policies focused on expanding requirements for and ensuring an adequate trial of evidence-based medical treatment before moving on to procedures and also narrowed the indications for ICA and PCI in stable CAD, endovascular intervention for lower extremity PAD, and sinus procedures for chronic rhinosinusitis. Stable CAD, lower extremity PAD, and chronic rhinosinusitis are the clinical conditions for which these procedures are nearly always performed in the outpatient setting. We obtained feedback on the new policies from practicing clinicians in Louisiana as well as Louisiana Medicaid managed care organization (MCO) leadership and sought public comments through an online government website posting.

### Policy Details

Prior to the policy intervention, there was no formal coverage policy within the Louisiana Medicaid FFS program for any of the procedures, and there was heterogeneity in the plan-specific policies of Louisiana Medicaid MCOs. The revised policy for ICA allowed coverage for selected specific conditions, including diagnosis of congenital heart disease, and assessment of patients with stable CAD who were candidates for PCI or coronary artery bypass graft surgery (eTable 1 in Supplement 1).^41^ PCI was considered medically necessary for patients who had intolerance to medications or who had persistent anginal symptoms despite reaching a target dose of at least 2 antianginal medications. Prior to the new policy enactment, only 1 Louisiana Medicaid MCO had plan-specific policies for these procedures, although it did not provide strict parameters about medical therapy.

The policy for endovascular intervention in PAD provided coverage for procedures in acute limb ischemia and chronic limb-threatening ischemia.^41^ Patients with intermittent claudication were required to have symptoms that impaired their ability to work or perform activities of daily living that persisted after a supervised or directed exercise program for at least 12 weeks and at least 6 months of optimal pharmacologic therapy with an antiplatelet medication, a statin, cilostazol, and antihypertensive medications titrated to achieve a goal blood pressure of 140/90 mm Hg or less. If patients smoked tobacco, there had to be at least 1 documented attempt at smoking cessation. A concurrent new policy covered up to 36 sessions of PAD rehabilitation (supervised exercise therapy) annually.

The policy for sinus procedures required patients to have at least 2 sinonasal symptoms for at least 12 weeks, despite use of saline nasal irrigation and nasal corticosteroids for 6 weeks and other pharmacotherapies (eg, biologics, antibiotics) if applicable.^41^ Patients also were required to have objective evidence of inflammation prior to receiving a sinus procedure.

### Policy Enactment

Louisiana Medicaid enacted the new evidence-based policies for ICA, PCI, and endovascular intervention on December 14, 2021, and for sinus procedures on February 10, 2022, within the Louisiana Medicaid FFS program.^41^ Louisiana’s Medicaid MCOs, which provide coverage for more than 90% of Medicaid members in Louisiana, were required to provide coverage that was no more restrictive than the enacted coverage policies after a 30-day adoption period (January 28, 2022, for ICA, PCI, and endovascular intervention^42^ and March 9, 2022, for sinus procedures^43^). Generally, MCOs retain the ability to provide coverage that is expanded over and above Louisiana Medicaid FFS coverage policies. Further, the coverage policy did not require the MCOs to implement prior authorization based on the coverage criteria. At the time of policy enactment, there were 5 Louisiana Medicaid MCOs; a sixth Medicaid MCO was added on January 1, 2023.^44^

### Policy Evaluation Outcomes

We evaluated the impact of these policies on procedural utilization (codes in eTable 2 in Supplement 1). The primary outcome was monthly outpatient utilization change for each procedure per 100 000 members, analyzed using Louisiana Medicaid claims for 12 months before and 18 months after policy enactment. The secondary outcome was monthly outpatient facility and professional expenditures for each procedure per 100 000 members.

### Medicaid MCO Policy Enactment

Louisiana Medicaid MCOs are required to update their policy manuals to align with the FFS policy within 30 calendar days of a change in FFS policy.^42,43^ We determined the extent to which Louisiana Medicaid MCOs enacted the policies by obtaining the text of their policies and comparing it with the Louisiana Medicaid FFS policy. To obtain each MCO’s policies, we first searched the MCO’s Louisiana Medicaid provider manual. If we could not find the policy there, we then searched plan-specific policies on the MCO’s website. We also assessed whether MCOs required prior authorization for the procedures.

### Statistical Analysis

We performed interrupted time series analyses of all 4 procedures by examining monthly procedural volume per 100 000 members in Louisiana Medicaid. Medicaid beneficiaries who were enrolled in Medicare or otherwise receiving third-party coverage within 1 year prior to the procedure, during the procedure month, or 6 months after the procedure were excluded from the analysis. The interrupted time series analyses assessed the magnitude and statistical significance of the differences in trajectories of the procedural volumes and costs with 12 points (1 per month) before and 18 points (1 per month) after the policy intervention (ie, the trigger event was the date of policy enactment, which varied by procedure). A separate linear regression model was fit to the preintervention data points and the postintervention data points, without any assumption about directionality. For comparison of temporal trends in procedures in Louisiana, particularly the COVID-19 pandemic, we analyzed another common procedure, colonoscopy, which did not undergo any changes in reimbursement policy. Colonoscopy is an invasive procedure performed for both screening and diagnostic purposes, and there is also evidence of low-value colonoscopy procedures.^45^ We compared the difference in trends between each procedure and colonoscopy between the 2 intervention periods, using a 3-way interaction of time × intervention period × procedure. A 2-tailed *P* < .05 was considered significant. With the goal of providing understanding of the patient population receiving procedures before and after policy enactment, we also provided demographic data; race and ethnicity were classified as available in Louisiana Medicaid data. We performed statistical analyses using Stata, version 16.1 (StataCorp LLC).

## Results

### Overview

At baseline (1 year prior to any policy enactment) in December 2020, there were 1 396 629 Louisiana Medicaid members; at the time of the first policy enactment in December 2021, there were 1 495 431 members; and at the final month of follow-up (September 2023), there were 1 548 265 members. Overall, 14 940 individuals (mean [SD] age, 43.5 [13.7] years; 53.0% female and 47.0% male; 38.0% Black, 46.5% White, 15.5% other or unknown race [including American Indian or Alaska Native, Asian, and Native Hawaiian or Other Pacific Islander]; 10.4% Hispanic or Latino and 89.6% not Hispanic or Latino) underwent one of the 4 procedures before policy enactment, and 20 882 (mean [SD] age, 43.3 [13.7] years; 52.6% female and 47.7% male; 37.2% Black, 46.3% White, 16.5% other or unknown race [including American Indian or Alaska Native, Asian, and Native Hawaiian or Other Pacific Islander]; 11.2% Hispanic or Latino and 88.8% not Hispanic or Latino) underwent a procedure after policy enactment.

**Figure 1.**
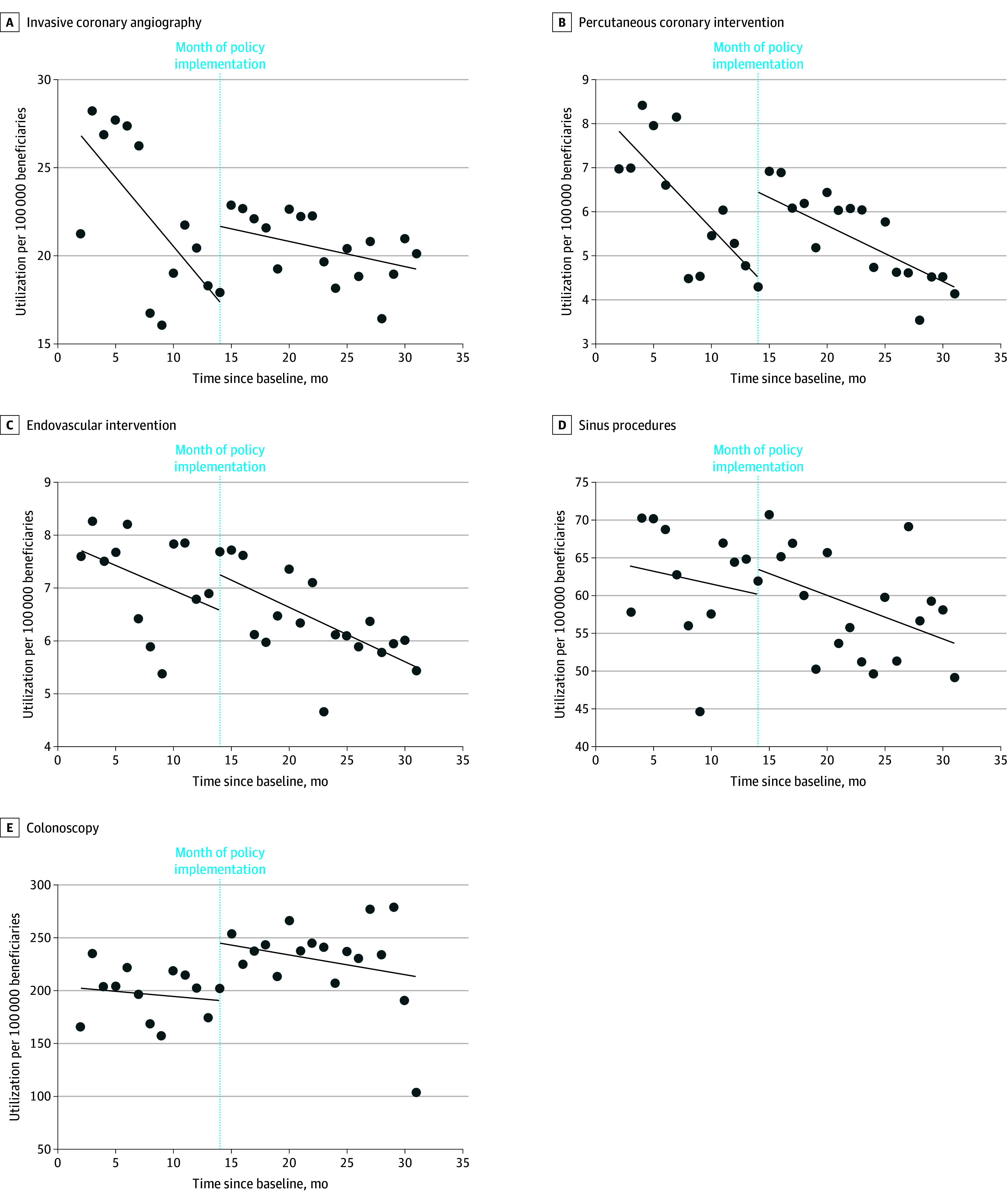
Trends in the Rates of Monthly Outpatient Utilization of Low-Value Procedures Before and After Enactment of Evidence-Based Coverage Policies in Louisiana Medicaid Sinus procedures refers to nasal endoscopy with balloon ostial dilation (balloon sinuplasty) and functional endoscopic sinus surgeries.

In summary, there was no significant decrease in observed monthly utilization per 100 000 members for any of the procedures (ICA, PCI, endovascular intervention, or sinus procedures) when using the fitted utilization trends in an interrupted time series analysis (Figure 1). Comparing differences between each procedure and colonoscopy in the utilization trends before and after policy enactment showed no significant differences for any of the procedures (Table 1). The 3-way time × intervention period × procedure vs colonoscopy interaction was not statistically significant for ICA, PCI, endovascular intervention, or sinus procedures. Results for the individual procedures are shown in the following sections.

**Table 1.** Trends in Monthly Outpatient Utilization per 100 000 Members Before and After Policy Enactment by Procedure

Procedure	Trend in monthly utilization rate, procedures per 100 000 members (95% CI)		Difference in monthly trends (95% CI)
	Preenactment	Postenactment	
Invasive coronary angiography	−0.79 (−1.34 to −0.23)	−0.14 (−0.33 to 0.04)	0.65 (0.06 to 1.23)
Percutaneous coronary intervention	−0.28 (−0.41 to −0.14)	−0.13 (−0.21 to −0.04)	0.15 (−0.01 to 0.31)
Endovascular intervention for peripheral arterial disease	−0.09 (−0.19 to 0.00)	−0.10 (−0.16 to −0.05)	−0.01 (−0.12 to 0.10)
Sinus procedures^a^	−0.34 (−1.64 to 0.95)	−0.57 (−1.09 to −0.05)	−0.23 (−1.61 to 1.15)
Colonoscopy	−0.93 (−4.74 to 2.87)	−1.81 (−7.23 to 3.59)	−0.88 (−7.67 to 5.91)

^a^ Sinus procedures refers to nasal endoscopy with balloon ostial dilation (balloon sinuplasty) and functional endoscopic sinus surgeries.

### ICA and PCI

#### Utilization

The mean (SD) age of patients receiving ICA was 51.7 (10.7) years before and 52.1 (10.6) years after policy enactment; 46.6% of patients were female prepolicy and 47.1% postpolicy (Table 2). In the 12 months before policy enactment, monthly ICA utilization decreased at a rate of −0.79 (95% CI, −1.34 to −0.23) procedures per 100 000 members, but in the 18 months after policy enactment, utilization decreased at −0.14 (95% CI, −0.33 to 0.04) procedures per 100 000 members (Figure 1). This slower decline in utilization of 0.65 (95% CI, 0.06-1.23) procedures was statistically significant (Table 1).

**Table 2.** Demographics of Louisiana Medicaid Members Receiving Procedures Before and After Policy Enactment

Characteristic	Invasive coronary angiography		Percutaneous coronary intervention		Endovascular intervention		Sinus procedures^a^	
	Prepolicy (n = 5702)^b^	Postpolicy (n = 7995)^c^	Prepolicy (n = 1301)^b^	Postpolicy (n = 1739)^c^	Prepolicy (n = 1366)^b^	Postpolicy (n = 1828)^c^	Prepolicy (n = 6571)^b^	Postpolicy (n = 9320)^c^
Mean (SD) age, y	51.7 (10.7)	52.1 (10.6)	54.0 (8.0)	54.2 (7.8)	54.5 (10.5)	54.2 (10.7)	32.1 (18.0)	31.6 (18.1)
Sex, No. (%)								
Female	2658 (46.6)	3767 (47.1)	516 (39.7)	706 (40.6)	578 (42.3)	814 (44.5)	4166 (63.4)	5693 (61.1)
Male	3044 (53.4)	4228 (52.9)	785 (60.3)	1033 (59.4)	788 (57.7)	1014 (55.5)	2405 (36.6)	3627 (38.9)
Race, No. (%)								
Black	2172 (38.1)	2953 (36.9)	429 (33.0)	562 (32.3)	608 (44.5)	774 (42.3)	2472 (37.6)	3489 (37.4)
White	2641 (46.3)	3695 (46.2)	652 (50.1)	875 (50.3)	576 (42.2)	787 (43.1)	3080 (46.9)	4308 (46.2)
Other and unknown^d^	889 (15.6)	1347 (16.8)	220 (16.9)	302 (17.4)	182 (13.3)	267 (14.6)	1019 (15.5)	1523 (16.3)
Ethnicity, No. (%)								
Hispanic or Latino	497 (8.7)	764 (9.6)	119 (9.1)	166 (9.5)	91 (6.7)	151 (8.3)	854 (13.0)	1253 (13.4)
Not Hispanic or Latino	5205 (91.3)	7231 (90.4)	1182 (90.9)	1573 (90.5)	1275 (93.3)	1677 (91.7)	5717 (87.0)	8067 (86.6)

^a^ Sinus procedures refers to nasal endoscopy with balloon ostial dilation (balloon sinuplasty) and functional endoscopic sinus surgeries.

^b^ The prepolicy intervention period was the 12 months prior to December 14, 2021, for invasive coronary angiography, percutaneous coronary intervention, and endovascular intervention and the 12 months prior to February 10, 2022, for sinus procedures.

^c^ The postpolicy intervention period was the 18 months following December 14, 2021, for invasive coronary angiography, percutaneous coronary intervention, and endovascular intervention and the 18 months following February 10, 2022, for sinus procedures.

^d^ Due to small cell sizes (<11), the race categories American Indian or Alaska Native, Asian, and Native Hawaiian or Other Pacific Islander are reported in this table under Other.

The mean (SD) age of patients receiving PCI was 54.0 (8.0) years before and 54.2 (7.8) years after policy enactment; 39.7% of patients were female prepolicy and 40.6% postpolicy (Table 2). In the 12 months before policy enactment, monthly PCI utilization decreased at a rate of −0.28 (95% CI, −0.41 to −0.14) procedures per 100 000 Medicaid members and in the 18 months after policy enactment at −0.13 (95% CI, −0.21 to −0.04) procedures per 100 000 members (Figure 1). The difference of 0.15 (95% CI, −0.01 to 0.31) procedures was not statistically significant (Table 1).

#### Spending

**Figure 2.**
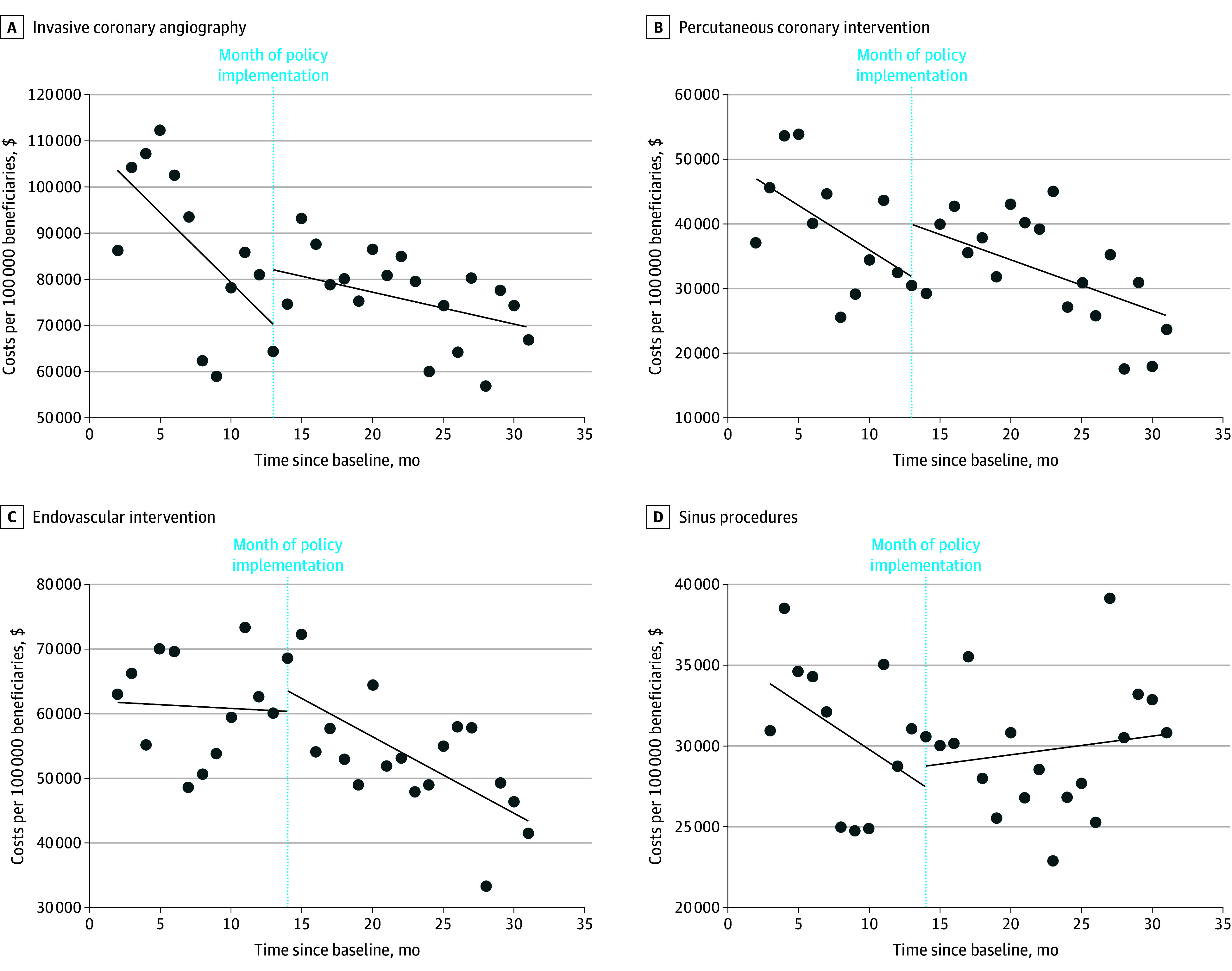
Trends in Monthly Spending for Low-Value Procedures Before and After Enactment of Evidence-Based Coverage Policies in Louisiana Medicaid Sinus procedures refers to nasal endoscopy with balloon ostial dilation (balloon sinuplasty) and functional endoscopic sinus surgeries.

In the 12 months before policy enactment, monthly spending for ICA decreased at a rate of −$3027 (95% CI, −$5770 to −$284) per 100 000 Medicaid members, and in the 18 months post policy enactment at −$680 (95% CI, −$1563 to $203) per 100 000 members (Figure 2). The difference of $2347 (95% CI, −$452 to $5145) was not statistically significant.

In the 12 months before policy enactment, monthly spending for PCI decreased at a rate of −$1386 (95% CI, −$2957 to $185) per 100 000 Medicaid members and in the 18 months after policy enactment at −$785 (95% CI, −$1430 to −$141) per 100 000 members (Figure 2). The difference of $601 (95% CI, −$1102 to $2304) was not statistically significant.

### Endovascular Intervention

#### Utilization

The mean (SD) age of patients receiving endovascular intervention was 54.5 (10.5) years before and 54.2 (10.7) years after policy enactment; 42.3% of patients were female prepolicy and 44.5% postpolicy (Table 2). In the 12 months before policy enactment, monthly endovascular intervention utilization decreased at a rate of −0.09 (95% CI, −0.19 to 0.00) procedures per 100 000 Medicaid members, and in the 18 months after policy enactment at −0.10 (95% CI, −0.16 to −0.05) procedures per 100 000 members (Figure 1). The difference of −0.01 (95% CI, −0.12 to 0.10) procedures was not statistically significant (Table 1).

#### Spending

In the 12 months before policy enactment, monthly spending for endovascular intervention decreased at a rate of −$121 (95% CI, −$1047 to $806) per 100 000 Medicaid members and in the 18 months after policy enactment at −$1172 (95% CI, −$1802 to −$543) per 100 000 members (Figure 2). The difference of −$1052 (95% CI, −$2169 to $66) was not statistically significant.

### Sinus Procedures

#### Utilization

The mean (SD) age of patients receiving sinus procedures was 32.1 (18.0) years before and 31.6 (18.1) years after policy enactment; 63.4% of patients were female prepolicy and 61.1% postpolicy (Table 2). In the 12 months before policy enactment, the monthly utilization of sinus procedures decreased at a rate of −0.34 (95% CI, −1.64 to 0.95) procedures per 100 000 Medicaid members and in the 18 months after policy enactment at −0.57 (95% CI, −1.09 to −0.05) procedures per 100 000 members (Figure 1). The difference of −0.23 (95% CI, −1.61 to 1.15) procedures was not statistically significant.

#### Spending

In the 12 months before policy enactment, monthly spending for sinus procedures decreased at a rate of −$575 (95% CI, −$1252 to $102) per 100 000 Medicaid members and in the 18 months after policy enactment, increased at a rate of $113 (95% CI, −$194 to $420) per 100 000 members (Figure 2). The difference of $688 (95% CI, −$69 to $1445) was not statistically significant.

### MCO Policy Enactment

Across Louisiana Medicaid’s 6 MCOs, 4 (67%) included the enacted policies for all procedures in their respective provider manuals (eTable 3 in Supplement 1). The sinus procedures coverage policy had the highest MCO adoption, with 5 (83%) MCO provider manuals including the enacted policy. Of the 2 MCOs that did not include all enacted policies in their provider manuals, 1 MCO had enacted less restrictive coverage policies across all procedures, while 1 MCO had not posted specific policies for the cardiovascular procedures to its provider manual.^42^

Of the 6 MCOs, 4 (67%) required prior authorization for at least 1 procedure, while no MCO required prior authorization for all of the procedures (eTable 4 in Supplement 1). ICA was the procedure for which prior authorization was most commonly required, with half of the 6 MCOs requiring prior authorization, whereas only 1 MCO each required prior authorization for PCI, endovascular intervention, and functional endoscopic sinus surgery.

## Discussion

This quality improvement study found that enactment of more restrictive evidence-based policies for 4 commonly performed medical device–based procedures was not associated with a reduction in the use of those procedures in Louisiana Medicaid. Consequently, there was no significant decline in expenditures for these procedures. Despite a process that included all stakeholders, including practicing clinicians in Louisiana and MCO leadership, to develop more rigorous evidence-based clinical coverage policies, there was no related decline in low-value use of these procedures. Our findings suggest a need for reflection on the multiple drivers of low-value procedure use and consideration of next steps for successful implementation of policies to reduce low-value care.

A possible explanation for the lack of a significant decrease in these procedures is that Medicaid MCOs often had less restrictive coverage policies than the agency-enacted coverage policy.^46^ These findings highlight the limitations of using FFS coverage changes to influence Medicaid utilization and the need for alignment between Medicaid FFS and Medicaid MCO coverage policy, as well as enforcement of coverage criteria; this is particularly important for the majority of states that predominantly administer their state Medicaid care through MCOs. Given the heterogeneity in policies across Louisiana’s Medicaid MCOs and different policies for other public and private insurers, it is difficult for clinicians to change patient care patterns according to variable insurer policies. However, it is an expectation that clinicians who contract with a payer (in this case, Louisiana Medicaid) are aware of these coverage policies; these coverage policies are publicly posted by the Louisiana Department of Health and Louisiana Medicaid MCOs. Consistent adoption of high-value coverage policies by all payers (including private insurers) would facilitate changes in practice patterns and drive evidence-based care.

Our findings that the policy enactment was not effective may suggest that in addition to discussing, revising, and posting a coverage policy, additional steps are essential to incentivize and ensure implementation. Two approaches used by insurers are prior authorization, in which payment is guaranteed if a procedure is approved in advance,^47^ and postpayment review, in which payment may be recouped if a procedure is found to not meet coverage criteria. However, use of prior authorization and postpayment review can be resource-intensive, complex, and burdensome.^47^ Louisiana’s Medicaid MCOs often did not use prior authorization; in fact, 2 MCOs did not require prior authorization for any of the procedures in our policies. While prior authorization often leads clinicians to change clinical decisions^48^ and has been shown to reduce low-value care,^49^ it is unpopular with clinicians. To address clinician abrasion, some insurers have introduced “gold-carding” based on history of appropriate use to exempt gold-carded clinicians from prior authorization requirements.^50^ Postpayment review is also not popular as it can penalize clinicians after the fact, when they may be unaware of the coverage issues. In addition, insurers do not universally use postpayment review, nor routinely perform it for all services. We did not have any specific data on postpayment reviews, as these are typically managed internally by agency and MCO program integrity departments. Other strategies, such as shared savings from lower procedural utilization, could be a helpful incentive for clinicians.^51^

Although we used a transparent, deliberative process and sought input from practicing physicians caring for Louisiana Medicaid members in developing coverage policies, clinicians may have chosen not to follow the new policies for these high-cost, invasive procedures that, to our knowledge, do not have published literature examining changes with updated clinical coverage policies. A FFS system with high reimbursement for procedures can drive overuse. Furthermore, overuse of PCI has occurred for many years^52^ and may be baked into the culture and practice of medicine, making change in practice much harder, particularly if it is not coupled with change in reimbursement policy. Additionally, despite overwhelming evidence that PCI does not reduce death or myocardial infarction in stable CAD, cardiologists still may believe that PCI could benefit patients for other reasons, such as alleviating patient anxiety and medicolegal considerations.^53^ Research has found that multiple interdependent factors, including the payment system, industry promotion, and a “more is better” culture, often lead to low-value care.^54^

Additionally, it is possible that the use of these 4 procedures was already evidence-based and, therefore, there was little opportunity to further reduce use. However, evidence shows that medical therapy is underused prior to use of all of these procedures.^16,24,25,32,55^ Finally, use of these procedures may have already been limited due to limited access to specialists in Louisiana Medicaid.

Given that prior literature has shown reduced utilization with prior authorization,^39^ it is possible that mandating Medicaid MCOs require prior authorization could be more likely to reduce low-value use of procedures. Any efforts to impose prior authorization should be balanced against considerations around maintaining adequate access to specialists within Medicaid MCOs. Additionally, clinician-focused strategies may be necessary. For example, performance metrics that document the proportion of patients receiving medical therapy prior to a device-based procedure could discourage low-value practices through peer comparisons.^56^ These could be sent privately to physicians in a plan and could include comparisons to the plan average. Alternatively, these data could be posted on the plan website or state medical association website. For example, published research comparing low-value services across hospitals, including identifying the specific hospitals, holds potential to reduce overuse.^57^ Regardless, it will be important to ensure that updated coverage requirements are not undermined. Research has shown that after initiatives to reduce rates of inappropriate PCIs, there was an increase in coding for unstable angina.^58^ Similarly, there has been a recent increase in Medicare beneficiaries receiving endovascular intervention with chronic limb-threatening ischemia (CLTI) codes, with corresponding decreases in claudication; however, rest pain can be subject to interpretation and was a partial driver of the increase in CLTI coding.^59^ It is likely that more intensive reviews will be needed to ensure that coverage requirements are not subverted in this way.

### Limitations

Our study should be considered in the context of its limitations. First, we did not have access to medical records and therefore could not assess whether reimbursed procedures met coverage criteria in the implemented policy. This limitation could obscure our findings if most procedures prior to enactment were already performed based on the coverage policy. Second, some of these data were collected during the COVID-19 pandemic, and procedural use could have been affected by the pandemic. Third, although there was heterogeneity in policy enactment across Louisiana Medicaid MCOs, there was an insufficient number of procedures at each MCO to meaningfully assess whether this heterogeneity affected utilization across each MCO or in comparison to FFS coverage. Fourth, any recoupments that occurred through postpayment review may not be reflected in our data, because such a review occurs after the service has been rendered and the claim has been paid. This lag may lead to an overestimate of procedures that would eventually be reimbursed. Fifth, our study is observational in nature and did not include randomization in its enactment. Accordingly, other factors outside of the policies could have impacted utilization. While we used colonoscopy as a comparator, other factors, such as changes in guidelines around screening colonoscopy age, could have affected utilization. Finally, our findings are limited to Medicaid in one state and may not generalize to other states or payers.

## Conclusions

The results of this quality improvement study indicate a need to continue to understand additional drivers of low-value use of device-based procedures and strategies to improve evidence-based care through effective implementation of changes in payment policies.

## Notes

Supplement 1. **eTable 1.** Details of Evidence-Based Clinical Coverage Policies Enacted by Louisiana Medicaid **eTable 2.** Current Procedural Terminology Codes for Procedures **eTable 3.** Enactment of Revised Policies by Louisiana Medicaid Managed Care Organizations **eTable 4.** Prior Authorization Requirements of Louisiana Medicaid Managed Care Organizations

Supplement 2.
Data Sharing Statement
