# Supplementary material for: Evaluation of Payer Policies to Reduce Low-Value Medical Device–Based Procedure Use
Source: JAMA Health Forum. 2025 Oct 31;6(10):e253898. doi: 10.1001/jamahealthforum.2025.3898 (PMC12579349; doi:10.1001/jamahealthforum.2025.3898)
Supplement: Supplement 1. — eTable 1. Details of Evidence-Based Clinical Coverage Policies Enacted by Louisiana Medicaid eTable 2. Current Procedural Terminology Codes for Procedures eTable 3. Enactment of Revised Policies by Louisiana Medicaid Managed Care Organizations eTable 4. Prior Authorization Requirements of Louisiana Medicaid Managed Care Organizations [file jamahealthforum-e253898-s001.pdf]

Supplementary Online Content

Dhruva SS, Tingley SR, Incze M, Neuhaus JM, Bachhuber MA, Redberg RF. Evaluation of payer policies to reduce low-value medical device-based procedure use. *JAMA Health Forum*. 2025;6(10):e253898. doi:10.1001/jamahealthforum.2025.3898

eTable 1. Details of Evidence-Based Clinical Coverage Policies Enacted by Louisiana Medicaid
eTable 2. Current Procedural Terminology Codes for Procedures
eTable 3. Enactment of Revised Policies by Louisiana Medicaid Managed Care Organizations
eTable 4. Prior Authorization Requirements of Louisiana Medicaid Managed Care Organizations

This supplementary material has been provided by the authors to give readers additional information about their work.

© 2025 Dhruva SS et al. JAMA Health Forum.

eTable 1. Details of Evidence-Based Clinical Coverage Policies Enacted by Louisiana Medicaid

Coverage Policy	Pertinent Details
Invasive Coronary Angiography	• Selected specific conditions, including diagnosis of congenital heart disease, and assessment of patients with non-acute stable coronary artery disease (CAD) who were candidates for percutaneous coronary intervention or coronary artery bypass graft surgery
Percutaneous Coronary Intervention	• For patients who had intolerance to medications or who had persistent anginal symptoms despite reaching a target dose of at least two anti-anginal medications<br>• Other coverage included for heart failure with reduced ejection fraction to treat ischemic cardiomyopathy, for left main stenosis declined for coronary artery bypass graft surgery, or type 1 myocardial infarction within past 3 months
Endovascular Revascularization for Peripheral Artery Disease	• For procedures in acute limb ischemia and chronic limb-threatening ischemia<br>• For patients with intermittent claudication whose symptoms their ability to work or perform activities of daily living that persisted after a supervised or directed exercise program for ≥12 weeks and at least 6 months of optimal pharmacologic therapy with an antiplatelet medication, a statin, cilostazol, and antihypertensive medications titrated to achieve a goal blood pressure ≤140/90 as well as at least one documented attempt at smoking cessation for patients who smoked tobacco<br>• Coverage of peripheral arterial disease rehabilitation (supervised exercise therapy) for up to 36 sessions annually
Sinus Procedures	• For patients with at least two sinonasal symptoms for at least 12 weeks, despite use of saline nasal irrigation and nasal corticosteroids for 6 weeks and other pharmacotherapies (e.g., biologics, antibiotics) if applicable, and objective evidence of inflammation

© 2025 Dhruva SS et al. JAMA Health Forum.

eTable 2. Current Procedural Terminology Codes for Procedures

Procedure	Current Procedural Terminology Codes
Invasive Coronary Angiography	93454, 93455, 93456, 93457, 93458, 93459, 93460, 93461
Percutaneous Coronary Intervention	92920, 92921, 92924, 92925, 92928, 92929, 92933, 92934, 92937, 92938, 92943, 92944
Endovascular Intervention	37220, 37221, 37222, 37223, 37224, 37225, 37226, 37227, 37228, 37229, 37230, 37231, 37232, 37233, 37234, 37235, 75710, 75716
Diagnostic Nasal Endoscopy	31231, 31233, 31235
Nasal/Sinus Endoscopy with Surgical Procedure	31237, 31238, 31239, 31240, 31241, 31253, 31254, 31255, 31256, 31257, 31259, 31267, 31276, 31287, 31288, 31290, 31291, 31292, 31293, 31294
Nasal/Sinus Endoscopy with Dilation of Sinus Ostia by Displacement of Tissue, Any Method, and Fluoroscopy if Performed	31295, 31296, 31297, 31298

© 2025 Dhruva SS et al. JAMA Health Forum.

eTable 3. Enactment of Revised Policies by Louisiana Medicaid Managed Care Organizations

Procedure	Revised Policy Enacted (Included in MCO Provider Manual), No. (%) N = 6	Revised Policy Not Included in MCO Provider Manual	
		Posted Policy Less Restrictive Than Revised Policy, No. (%), N = 6	No Policy Available Online[a], No. (%) N = 6
Invasive Coronary Angiography	4 (67%)	1 (17%)	1 (17%)
Percutaneous Coronary Intervention	4 (67%)	1 (17%)	1 (17%)
Endovascular Revascularization for Peripheral Artery Disease	4 (67%)	1 (17%)	1 (17%)
Nasal Endoscopy with Balloon Ostial Dilation	5 (83%)	1 (17%)	0 (0%)
Functional Endoscopic Sinus Surgery	5 (83%)	1 (17%)	0 (0%)

[a]Coverage policy not posted online in MCO provider manual or in the MCO's plan-specific policies for Louisiana Medicaid.
Abbreviations: MCO, managed care organization.

© 2025 Dhruva SS et al. JAMA Health Forum.

eTable 4. Prior Authorization Requirements of Louisiana Medicaid Managed Care Organizations

Procedure	Managed Care Organizations Requiring Prior Authorization[a], No. (%) N = 6
Invasive Coronary Angiography	3 (50%)
Percutaneous Coronary Intervention	1 (17%)
Endovascular Revascularization for Peripheral Artery Disease	1 (17%)
Nasal Endoscopy with Balloon Ostial Dilation	2 (33%)
Functional Endoscopic Sinus Surgery	1 (17%)

[a]Prior authorization was considered required for a procedure if the provider manual indicated that prior authorization was required for the service or if all Healthcare Common Procedure Coding System (HCPCS) codes associated with the procedure were included in the organization's prior authorization code list.

© 2025 Dhruva SS et al. JAMA Health Forum.
