# Supplementary material for: Evaluation of Payer Policies to Reduce Low-Value Medical Device–Based Procedure Use
Source: JAMA Health Forum. 2025 Oct 31;6(10):e253898. doi: 10.1001/jamahealthforum.2025.3898 (PMC12579349; doi:10.1001/jamahealthforum.2025.3898)
Supplement: Supplement 2. — Data Sharing Statement [file jamahealthforum-e253898-s002.pdf]

Data Sharing Statement

Dhruva. Evaluation of Payer Policies to Reduce Low-Value Medical Device–Based Procedure Use. *JAMA Health Forum*. Published October 31, 2025.
doi:10.1001/jamahealthforum.2025.3898

Data
Data available: No

Additional Information
Explanation for why data not available: Data accessed under Data Use Agreement with the Louisiana Department of Health, Bureau of Health Services Financing (Medicaid) and the University of Louisiana at Monroe.
